# New daily persistent headache with a thunderclap headache onset and complete response to Nimodipine (A new distinct subtype of NDPH)

**DOI:** 10.1186/1129-2377-14-100

**Published:** 2013-12-23

**Authors:** Todd D Rozen, Jennifer L Beams

**Affiliations:** 1 Department of Neurology, Geisinger Health System, Geisinger Headache Clinic, MC 37-32, 1000 East Mountain Blvd, Wilkes-Barre, PA 18711, USA

**Keywords:** New daily persistent headache, Thunderclap headache, Vasospasm, Nimodipine, Reversible cerebral vasoconstriction syndrome, Acalculia

## Abstract

At present new daily persistent headache is just a group of conditions that are connected based on the temporal profile of their mode of onset. If new daily persistent headache is a true distinct syndrome like migraine then we need to start to define subtypes that have specific effective treatments such has been noted for migraine sub-forms. We present what we believe is the first recognized subtype of new daily persistent headache that which starts with a thunderclap headache onset. A patient presented with a 13 month history of a daily headache from onset which initiated as a thunderclap headache along with persistent acalculia. All neuroimaging studies for secondary causes were negative. Nimodipine rapidly and completely alleviated her headache and associated neurologic symptoms. We propose that this subtype of new daily persistent headache is caused by a very rapid increase in CSF tumor necrosis factor alpha levels leading to cerebral artery vasospasm with a subsequent thunderclap headache, then continuous or near continuous cerebral artery vasospasm leading to a persistent daily headache. Nimodipine which not only inhibits cerebral artery vasospasm but also tumor necrosis factor alpha production appears to be a specific treatment for this distinct subtype of new daily persistent headache.

## Background

The lack of consensus on a clear definition of new daily persistent headache (NDPH) and the fact that we are still searching for adequate therapy reflects on how little we know about this syndrome [1]. Is NDPH a group of conditions that just happen to all start daily from onset and have little else in common including no shared underlying pathogenesis or is there some distinct cortical change that causes a daily headache from onset in all patients with NDPH although the mechanism by which this cortical phenomena is triggered may be different for individual patients (post-infectious, post-surgical for example)? If NDPH is a distinct all-encompassing syndrome like migraine then we need to define sub-forms that may have specific effective treatments such has been noted for migraine with aura for example [2]. The end result of this will be improved patient satisfaction. The presented case appears to be the first documented distinct subtype of NDPH: NDPH with a thunderclap headache onset. The treatment for this distinct case of NDPH is nimodipine.

## Case presentation

A 46-year-old woman presented with a daily headache from onset that began 13 months prior. The daily headache started as a thunderclap headache. She was playing bingo and she suddenly developed the worst headache of her life which peaked immediately to 10/10 in intensity without latency. This was associated with vomiting and dizziness. The peak headache lasted for approximately 24 hours and thereafter she was left with a persistent lower grade headache which never waned. She denied having any further thunderclap headaches. With the original thunderclap headache she did not seek emergency attention. She saw her PCP the following day and had a brain MRI with and without gadolinium within 7 days which was reportedly normal with no evidence of subarachnoid blood. No lumbar puncture was ever completed. Her persistent daily headache was typically of moderate severity (4-5/10 VAS) and was localized to the right occipito-nuchal region. There was never any pain free time and she experienced intermittent associated symptoms including nausea and photophobia. She never experienced any cranial autonomic associated symptoms. The patient denied any headache triggering or alleviating maneuvers. She could not identify any precipitating event just prior to the onset of her daily persistent headache including no viral infection, stressful life event and she had not had any surgical procedures. She was on citalopram at the time of headache onset although she denied the use of marijuana, ecstasy or pseudoephedrine. In addition to head pain the patient also complained of having issues with numbers including doing simple addition and subtraction, recognizing the order of numbers and even recognizing certain numbers. She had to change her pin number multiple times because she could not place the numbers in the correct order on a keypad. She was even unable to copy down telephone numbers. She worked as a pharmacist and was actually very proficient in mathematics so the issue with numbers and calculations was very troubling to her and she was at risk of losing her job. Her acalculia (part spatial, part anarithmetria) started the same day as her daily headache. Her neurologic examination on presentation to the headache clinic was intact including a normal neurovascular examination, normal language examination, normal ability to read and write, but on serial 7 s testing she could not get below 93 when starting at 100. She also could not copy numbers in a correct order when they were presented to her verbally. In addition she had right greater occipital notch and occiput based pain to palpation although she stated this palpable tenderness was not her true pain which was “deep” to the skull. As the patient already had brain neuroimaging, cerebral vessel imaging was ordered to complete the evaluation for thunderclap headache and this included CT angiography of the head and neck vessels as well as brain venography and all studies were negative including no evidence for aneurysms, vessel dissection, vasospasm or thrombosis. An EEG was also completed and this was a normal study. Prior failed therapies which were minimal before coming to the dedicated headache clinic included near daily over the counter analgesics which were minimally effective, topiramate which lowered daily headache intensity but did not provide any pain free time and did not alter calculation issues, and oral prednisone which reduced but did not eliminate her headache (of note sedimentation rate and c-reactive protein were normal). The patient was given a diagnosis of NDPH as she met ICHD-3 beta criteria [3] which began as a thunderclap headache. Her headache did not meet criteria for hemicrania continua although it was one-sided. She also had persistent acalculia. As nothing was noted on imaging the authors surmised that this may be a syndrome of persistent vasospasm and possibly even reversible cerebral vasoconstriction syndrome (RCVS) induced by citalopram and the acalculia was caused by persistent oligemia to the cortex. Citalopram was discontinued. Nimodipine was started for preventive therapy at a dose of 30 mg PO BID and within 4 days of starting therapy the patient became headache free. After 3 weeks on nimodipine her acalculia resolved. On re-evaluation two months after her initial visit she remained pain free. After 4 months on medication the patient decided to taper off her nimodipine and her daily headaches returned almost immediately although the calculation issues remained resolved. Nimodipine was restarted at 30 mg bid and within 3 days her headaches again ceased. This verified that the nimodipine had cured her headaches and it was not just being off citalopram. Over time she has been able to reduce the dose to 30 mg one time per day and still she remains headache free. She is hesitant to come off the medication in fear the pain will return. She has been followed for almost one year.

## Conclusions

The question arises does this patient have a distinct subtype of NDPH and if so was it caused by continuous vasospasm and did the vasospasm lead to oligemia to specific regions of the cortex which led to acalculia? Based on the patient’s response to nimodipine (a known inhibitor of cerebral artery vasoconstriction) this is a distinct possibility. Isolated acalculia without agraphia or alexia has been attributed to lesions mainly involving the dominant parieto-temporal cortex but has also been associated with injury to the medial frontal cortex, caudate nucleus, internal capsule, putamen and can also involve the non-dominant hemisphere when there is impairment with spatial organization of numbers which this patient displayed [4]. No ischemia was noted on this patient’s brain MRI but that does not mean that one or more of these cortical regions was not altered from persistent oligemia; it just means that changes could not be seen by conventional neuroimaging. We wanted to do SPECT or PET imaging to better asses for changes in cortical blood flow, but her medical insurance denied coverage for this testing.

If vasospasm is the probable cause of NDPH with a thunderclap headache onset then one needs to then question if this subtype of NDPH is a prolonged subform of RCVS and thus an overlap disorder that could be placed under two ICHD sub-headings? In most instances RCVS is a self-limited syndrome of individual thunderclap headaches that by ICHD-3 beta criteria [3] does not last longer than one month in duration [4]. However, Hastriter et al. [5] in a Mayo Clinic series identified 16 patients with RCVS of whom six patients developed chronic daily headache when followed over and average of 99 weeks. The number of these patients who had a presentation of daily persistent headache after their initial thunderclap headache was not stated in the published abstract, although five patients were documented to have a continuous headache in between their thunderclap headaches, thus a possible NDPH type picture. Treatment for these patients was also not reported. In the Hastriter et al. [5] population there was documented resolution of vasoconstriction around 20 weeks after symptom onset, thus the cause of the patient’s chronic headaches was not felt to be from vasospasm and thus not known. It is possible our patient was an outlier case of prolonged RCVS and the result of persistent cerebral artery vasospasm that lasted for more than one year’s duration, which was not picked up on CT angiography, did not cause ischemia on MRI, but did cause enough oligemia leading to acalculia and head pain and nimodipine alleviated the vasospasm and thus headache and neurologic symptoms; but this can only be hypothesized. Conventional angiography would have been helpful as it could have better identified vasospasm but it was not completed as the patient so quickly responded to treatment and we did not want to subject her to another test which conceivably had a stroke risk as a potential complication. What possibly goes against the hypothesis of very prolonged vasospasm for our patient is the data of Hastriter et al. [5] which showed self-limited vasoconstriction even in those RCVS patients who developed chronic daily headache. What also is atypical for a diagnosis of RCVS is the fact that our patient only experienced a single thunderclap headache while most patients with RCVS have recurrent quick peaking headaches. If indeed our patient had a daily persistent headache secondary to RCVS or a sequela of some other underlying phenomenon but the testing for secondary causes is all negative then classification of this disorder as a primary headache syndrome demonstrates some of the limitations of the current ICHD criteria [3]. This patient’s headache did indeed meet the ICHD-3 beta criteria [3] for primary NDPH as no secondary cause was found on neuroimaging but with response to nimodipine there is high clinical suspect the headache was at least partially secondary to cerebral artery vasospasm. The duration of time from onset of symptoms to when the patient was seen at a dedicated headache clinic also complicates issues with classification, as secondary causes which may have been noted on imaging or other testing if ordered properly at the onset of symptoms, may not be present when ordered many months later, thus a correct secondary diagnosis cannot be made at the time when a patient is evaluated by a headache specialist. For NDPH in particular where the underlying pathogenesis has not been elucidated and in which most patients have negative studies, classifying any form as primary NDPH is actually difficult if not impossible, when all cases may have a secondary underlying cause like the proposed cerebral artery vasospastic issue in our case patient.

Nimodipine has shown very little effect in preventive migraine studies [6]. Nimodipine has however shown efficacy in the treatment of non-migrainous vascular headaches in individuals with chronic brain ischemia especially those with more significant headache intensity [7]. The proposed mechanism of action for headache control was inhibition of cerebral vasoconstriction. Nimodipine has not previously been documented to have been tried in NDPH [1]. There is recent animal data indicating that nimodipine can decrease the expression of CGRP in the trigeminal nucleus caudalis of rats [8]. As CGRP is a known trigger of migraine attacks it conceivably may also have a role in the pathogenesis of NDPH, another trigeminal based pain syndrome [9]. Why nimodipine however would be more effective for NDPH than for migraine would be hard to understand if its effect was solely by modulating CGRP expression. Nimodipine has also been shown to inhibit glial activation and tumor necrosis factor (TNF) alpha production in several animal models and this could be one mechanism by which it may work in NDPH [10,11]. One of the authors (TDR) has previously shown that patients with NDPH had elevation of CSF TNF alpha levels and thus TNF alpha was a probable inciting cytokine in the pathogenesis of this syndrome, most likely thru its ability to produce CNS inflammation [12]. TNF alpha also is a vasoconstrictive cytokine and thus could have a distinct triggering potential in NDPH with a thunderclap headache onset [13]. In mice TNF alpha is able to evoke a direct vasoconstrictor effect on isolated cerebral vessels through Rac-1 activation [13]. In humans there is anecdotal evidence that TNF alpha plays a role in cerebral artery vasospasm as demonstrated by peak elevations of CSF TNF alpha levels on the days corresponding to peak vasospasm time in patients who have had a subarachnoid hemorrhage and in those showing elevated flow velocities on transcranial doppler after a subarachnoid bleed [14]. We can thus hypothesize that the syndrome of NDPH with a thunderclap headache onset is caused by a very rapid increase in CSF TNF alpha levels leading to cerebral artery vasospasm with a subsequent thunderclap headache, then continuous or near continuous cerebral artery vasospasm leading to a persistent daily headache and possibly other neurologic sequelae. To prove this hypothesis a csf analysis would need to be completed at the time of NDPH and thunderclap headache onset and it would need to demonstrate TNF alpha levels higher than that noted in the author’s prior csf study in typical NDPH without a thunderclap headache onset. This could not be done in this patient as we met her 13 months after symptom onset. Nimodipine theoretically works in this specific subtype of NDPH in a dual fashion by directly inhibiting cerebral artery vasospasm (calcium channel effect) and also by inhibiting TNF alpha production which not only prevents cerebral artery vasospasm, but in addition blocks the TNF alpha cytokine inflammatory response a proposed trigger of NDPH pain.

As NDPH is recognized as one of the most treatment refractory of all chronic headache conditions, documenting treatment responsive subtypes will be beneficial to both patients and providers. How rare is the subtype of NDPH with a thunderclap headache onset is unknown at present. The authors can state that in their practice which has a certain bias for NDPH patients, that this is the only patient who has fit this exact clinical presentation over the past year. Two other patients had a similar presentation of daily headache after a thunderclap headache onset with complete relief of their syndrome with nimodipine, but both had multiple thunderclap headaches during the first month of onset of their NDPH and thus were felt to be more consistent with a variant of RCVS and in line with the Hastriter et al. [5] reported patients, while our above reported case subject had only a single thunderclap headache at the outset of her daily persistent headache with no other recurrent thunderclaps, which distinguished her clinical presentation and placed it more in line in our opinion with an NDPH variant. It is certainly important when taking an NDPH history to ascertain what was the presentation of the original headache because if it was thunderclap then the provider should not only do the proper secondary work-up (neuroimaging should include MR angiography of the head and neck vessels, MR venography, MRI brain with and without gadolinium and a lumbar puncture if the patient is seen in the acute headache setting) but they should now think of nimodipine as a possible treatment option, which at present has not been part of the typical NDPH treatment protocol. There are two other case reports in the literature that should be mentioned which have some similarities to the present case report. A case of juvenile myoclonic epilepsy published by one of the authors (TDR) in which treatment of an associated neurologic condition in this case epilepsy with lamotrigine led to alleviation of NDPH which mimics our present case in which treatment of probable underlying vasospasm and TNF alpha elevation with nimodipine alleviated co-associated NDPH [15]. A second case report where NDPH was preceded by an acute clinical event also known to be associated with an elevation of TNF alpha, in this case heat stroke, and with no other secondary medical issues noted [16]. In conclusion the hope is that this present case will stimulate others to publish cases of NDPH with a thunderclap headache onset to determine how rare or common this presentation of NDPH truly is.

## Consent

Patient gave signed consent for case report.

## Abbreviations

(NDPH): New daily persistent headache; (RCVS): Reversible cerebral vasoconstriction syndrome; (TNF): Tumor necrosis factor.

## Competing interests

The authors declare that they have no competing interests.

## Authors' contributions

Both authors equally prepared in the drafting and finalizing of this manuscript. Both authors read and approved the final manuscript.
